# Postpartum check-ups with general practitioners in Norway: a cross-sectional survey of attendance, content and patient satisfaction

**DOI:** 10.1186/s12875-025-02992-x

**Published:** 2025-10-07

**Authors:** Christine Agdestein, Gunnhild Åberge Vie, Ingrid Baasland, Julie Horn, Bente Prytz Mjølstad

**Affiliations:** 1 General Practice Research Unit, Department of Public Health and Nursing, Norwegian University of Science and Technology, Trondheim, Norway; 2 Department of Obstetrics and Gynecology, Levanger Hospital, Nord-Trøndelag Hospital Trust, Levanger, Norway; 3 Yale School of Public Health, New Haven, CT 06510 USA; 4 HUNT Research Center, Department of Public Health and Nursing, Norwegian University of Science and Technology, Levanger, Norway; 5 Baasland Clinic AS, Trondheim, Norway; 6 Section for Cervical Cancer Screening, Cancer Registry of Norway, Norwegian Institute of Public Health, Oslo, Norway

**Keywords:** General practice, Family medicine, Maternal health, Patient satisfaction, Postpartum care, Primary health care, Questionnaire survey, Women’s health

## Abstract

**Background:**

A postpartum check-up regarding women’s health and well-being is recommended four to eight weeks after giving birth. We aimed to study postpartum check-ups with General Practitioners (GPs) in Norway. We assessed attendance, importance of key topics, check-up content and patient satisfaction.

**Methods:**

We conducted a cross-sectional countywide survey among women who gave birth in the hospitals in Nord-Trøndelag in Norway from 2021 to 2022. Women reported whether they attended the postpartum check-up, and reasons for non-attendance. Women who attended with their GP reported the importance of key topics, which topics were discussed, and which physical examinations were performed. We assessed patient satisfaction and identified factors associated with satisfaction using logistic regression analyses.

**Results:**

Out of 1,119 invited women, 351 (31.4%) responded. Among the responders, 254 (72.4%) attended the postpartum check-up; 211 with the GP, 40 with a municipal midwife and 3 in the hospital. Women with pre-existing conditions and adverse pregnancy outcomes did not attend more frequently than those in good health. Reasons for not attending included not having a regular GP, feeling check-up was unnecessary, low satisfaction with previous check-ups and lack of knowledge about check-ups. Labour, contraception, breastfeeding and genital birth injuries were rated as the most important topics. These topics, in addition to mental health, were most frequently discussed. Check-ups covered a median of eight topics, and 34% included a gynecological examination. More than half of the women (55%) were satisfied with the check-up with the GP. For each additional topic discussed, the odds of satisfaction increased by 38% (OR 1.38, CI 1.3–1.5). Discussing labour (OR 5.9, CI 2.1–16.3) and having a gynecological examination (OR 7.6, CI 2.4–23.6) were strongly associated with being satisfied with the check-up.

**Conclusion:**

More than one in four women missed the postpartum check-up, losing a key opportunity for support and health promotion. The majority of women were satisfied with the GP check-up. Satisfaction was strongly associated with the number of topics addressed, discussing labour experiences, and including a gynecological examination. Adopting a personalized and comprehensive approach could encourage higher attendance and increase patient satisfaction.

**Supplementary Information:**

The online version contains supplementary material available at 10.1186/s12875-025-02992-x.

## Introduction

The purpose of postpartum care is to ensure the survival, health and well-being of the mother and child, supporting parents and promoting nurturing care for the newborns [1, 2]. The World Health Organization (WHO) and the National Institute for Health and Care Excellence (NICE) recommend comprehensive follow-up of mothers after childbirth, including postpartum check-ups [1, 2]. The recommended timing of the postpartum check-up varies internationally. The WHO advises the check-up at six weeks postpartum, tailoring the timing to meet each woman’s health and needs [1]. NICE recommends the check-up at six to eight weeks postpartum [2], while the American College of Obstetrics and Gynecology recommends an initial contact with an obstetrician within the first three weeks postpartum, followed by a comprehensive check-up no later than 12 weeks after birth [3]. Limited evidence on optimal timing of postpartum care contacts [1] and differences in health care systems may contribute to inconsistent recommendations. The scope of the postpartum check-up is to support mothers, review the pregnancy and labour experience and address emotional well-being, breastfeeding, medical complications, mental and sexual health, contraception, coping strategies, and support networks [1, 2]. It is also an opportunity to discuss future health and lifestyle, especially for women with adverse pregnancy outcomes associated with long-term risk of cardiometabolic disease [4]. When assessing the quality of postpartum care, WHO recommends assessment of women’s satisfaction with the health services and women’s opportunities to discuss their concerns and preferences [5]. Patient-reported experiences are correlated with clinical safety and effectiveness [6].

Women seek a positive postpartum experience, which entails building confidence as a mother, navigating physical and emotional challenges, adapting in relationships and restoring health [7, 8]. Many feel unprepared for the physical and emotional symptoms that may occur after childbirth, and seek more information from healthcare professionals about how common symptoms are, their duration, and resources to cope [8, 9]. Mothers and general practitioners (GPs) view general practice as an important source of postpartum care for women and their families [10, 11]. Studies from England and Australia found that women wanted to discuss a broad range of topics with their GPs, including physical health and recovery, mental well-being, infant feeding, contraception, health promotion, and personal concerns [10, 12]. The postpartum period is a window of opportunity for health promotion provided by GPs [13].

Studies in the United States (U.S.), United Kingdom (UK) and Sweden, report that 72–90% of postpartum women attend the check-up [14–17]. Attendance is influenced by socioeconomic factors, even in countries with universal health care [17, 18]. While several studies have explored individual components of postpartum care, few have examined the full content of postpartum check-ups from women's perspectives [10, 17]. A representative study investigating postpartum experiences of U.S. mothers found that women were most likely to receive information about birth control (57% of respondents) and least likely to be informed about sexual health (30% of the respondents) [14]. Women who attended midwives most often received information about birth control, while women who attended family physicians most often received information about postpartum depression, healthy eating, exercise and changes in sexual health. Women attending obstetricians were least likely to be informed about these topics [14].

From physicians’ point of view, American obstetricians and family physicians prioritized depression, breastfeeding, labour complications and family planning during check-ups. Family physicians also commonly perform gynecological examinations [19]. English GPs reported that they most frequently addressed contraception, mental health and physical conditions among 26 clinical items from NICE guidelines [12]. GPs describe that they have a holistic approach to health and health promotion [11], supporting the “transition into parenthood” [19].

A 2024 U.S. report found that 24% of women rated their postpartum care as less than adequate [20]. In an Australian study, mothers reported inconsistencies in the scope and quality of the check-ups [10]. Despite these insights, there is a notable lack of quantitative studies specifically examining patient satisfaction with postpartum check-ups. The Norwegian Institute of Public Health assessed patient experiences from birth and postnatal care in 2013 and 2016, but did not include postpartum check-ups with GPs [21, 22], even though they are recommended for all mothers in the Norwegian national guideline for postpartum care [23]. The proportion of women attending postpartum check-ups in Norway remains unknown, as do potential social and health-related disparities in attendance. Furthermore, little is known about the actual content of these check-ups and which topics mothers consider as important.

In England postpartum check-ups are conducted by GPs [2, 24], and in the U.S. most women attend an obstetrician [25, 26]. While postpartum care in Norway is provided by GPs, midwives, nurses and obstetricians, their roles, areas of expertise and work context differ. The postpartum check-up has traditionally been the responsibility of GPs, and this study specifically examines the care delivered by GPs during postpartum check-ups.

We aimed to study postpartum check-ups with GPs from women’s perspectives, assessing attendance, the importance of key topics, the content of the check-ups, and patient satisfaction.

## Materials and methods

### Setting: Norwegian postpartum health care

In Norway, every resident has the right to choose a designated, regular GP, who provides continuity of care before, during and after pregnancy. Almost all women give birth in public hospitals and have an average postpartum hospital stay of 2.6 days before returning to primary care [27]. The national guideline for postpartum care recommends a home visit by a municipal midwife 1–2 days after discharge, a public health nurse visit after 7–10 days, followed by a postpartum check-up four to six weeks after birth [23]. Women with adverse pregnancy outcomes or chronic diseases are recommended to attend their GP sooner [23]. The guideline does not provide a comprehensive, structured list of topics to be covered, but recommends assessing physical and mental health, contraception, uncovering issues that need to be followed further and supporting the woman. Traditionally, the postpartum check-up takes place with the GP. In some municipalities, women can choose to have the check-up with a midwife. Consultations with GPs typically last 15–20 min, including time for documentation, whereas midwives often offer 30 min consultations. The postpartum check-up is free of charge, covered by the Norwegian universal insurance program.

### Study population

All women who gave birth to one or more liveborn children in the two hospitals of Nord-Trøndelag Health Trust (HNT) from 01.07.2021–30.06.22 were eligible for inclusion. Potential study participants were identified from the patient-administrative system by ICD-10 codes Z37.0 “Single birth, living child”, Z32.2 “Twin birth, both living children”, Z37.5 “Other multiple births, all living children”. The invitation to the survey was sent using eFORSK, a web-based application maintained by Central Norway Regional Health Authority’s IT department. Of the 1,144 eligible women, 25 women were excluded due to technical issues with the distribution of the invitation. The remaining 1,119 women received an invitation letter and a link to the digital questionnaire. Women were invited two to six months after childbirth, so that they would have had time to complete their postpartum check-up and still be able to recall the experience. Approximately one month later, one reminder was sent to everyone who had received the invitation. The questionnaire was made in the online survey tool “Nettskjema”, operated by the University of Oslo. Consent for participation was given by submitting the survey. The survey was anonymous, did not collect any person-identifiable information and responses could not be traced back to the respondent. Nord-Trøndelag is representative of the Norwegian population, except a low number of immigrants and the lack of a large city [28]. Inviting all mothers who gave birth in the region allowed comparison of the respondents to the demographic characteristics of the region and country population.

### Questionnaire design and variable classification

The questionnaire was in Norwegian only. An English translation is provided in the additional file (Questionnaire, Additional File 1). The questionnaire had three parts. All respondents reported demographic and background data, sources of information about the postpartum check-up, whether they attended a postpartum check-up with a GP, and reasons for non-attendance. The second part regarding pregnancy is outside the scope of this article. The third part addressed postpartum check-ups with GPs specifically, examining the importance of key topics, the content of the check-ups, and patient satisfaction.

As health-related background data, women reported whether they had chronic medical conditions, specified as high blood pressure, thyroid disease, diabetes, kidney disease, depression, anxiety, post-traumatic stress disorder, arthritis or other. Women indicated if they had been notified that they had a high-risk pregnancy. Complications during labour were specified as preeclampsia, major bleeding requiring surgery or blood transfusion, planned or emergency cesarean section, baby needed monitoring/treatment in the neonatal unit, birth before 37 weeks of pregnancy or other.

Women who had attended postpartum check-ups with GPs scored the importance of 22 key topics on a five-point Likert scale from “Not at all important” to “Very important”. The topics were derived from guidelines for postpartum care [1, 23] and the authors' clinical experience. Next, we asked whether each topic was discussed. “Do not know” was combined with “no topic was not discussed”. Due to high correlations between the topics “low mood”, “worry and rumination” and “postpartum depression” (Spearman’s rho 0.52–0.64), we combined them in a category called “mental health”, indicating whether either of the topics were discussed. For importance, we calculated the average score and rounded it to the nearest integer. “Physical activity” and “diet” were also correlated at 0.55 and were similarly combined into “lifestyle”. Therefore, subsequent analyses and figures include 19 topics. The variance inflation factor was tested using a linear model with the dichotomous overall satisfaction as the outcome and was below 5 for all topics. Women reported whether the following examinations were performed: blood pressure measurement, anemia assessment, gynecological examination, abdominal examination and others.

We included seven Patient Reported Experience Measurements (Questionnaire items 50–56, Additional File 1) from validated questionnaires developed by The Norwegian Institute of Public Health [29]. The items have previously been used in studies of GPs [30], hospital physicians [31] and maternity and postnatal wards [21]. The response format was a five-point Likert scale. The main patient satisfaction outcome was “Overall satisfaction with the check-up”. Responses were dichotomized into 'Satisfied' (to a great and very great extent) and 'Not satisfied' (somewhat, to a small extent, not at all). Last, we asked for free-text suggestions for improvements that could increase satisfaction.

We piloted the questionnaire with three patients from general practice and three physicians. They evaluated the completion time, technical feasibility, relevance of items and whether it was easy to understand. After the pilot, questions 50–56 were added.

### Statistical analysis

Categorical variables are described using frequencies with percentages. For comparison of responders to the female population in Trøndelag county and Norway, we present data on education levels from Statistics Norway [32], and age and parity from the Medical Birth Registry of Norway [33].

We used logistic regression to analyze the association between attending a postpartum check-up with either a GP, a midwife or a hospital, and the following predictor variables: Age, education, parity, self-rated health, physical and mental chronic illness, high-risk pregnancy, labour complications, mode of delivery, regular use of medication during pregnancy, municipality inhabitants, knowing that check-up is free and early contact with GP. Each model included one predictor adjusted for age and education.

We used logistic regression to analyze the association between being satisfied with the check-up overall and whether each listed topic/examination was covered during the check-up, adjusting for age, parity, physician gender and all other topics and examinations covered.

We assessed overall satisfaction by the number of topics covered, first using median and interquartile range (IQR). We thereafter used logistic regression to analyze the association between being satisfied with the check-up overall and the number of topics discussed, adjusting for gynecological examination. Based on this regression model, we used the predict function in R to estimate the predicted probabilities of being satisfied depending on the number of topics discussed under two conditions: with and without a gynecological examination. Sensitivity analyses showed that adjusting for additional variables (age, parity, GP gender, duration of patient-GP continuity, blood pressure measurement and abdominal examination) resulted in only minor changes in effect estimates and goodness-of-fit. We therefore chose the parsimonious model, which also gave the most conservative odds ratio predictions.

We used logistic regression to analyze the association between being satisfied with the check-up overall and physician gender, fitting first a univariate model, then a model adjusted for the number of topics and examinations covered. Odds ratios (ORs) are presented with 95% confidence intervals (CI), adjusted ORs are denoted adjOR.

Two researchers (CA and GV) independently reviewed the free-text answers, identified recurring categories, and quantified them. The results were compared and discussed until consensus was reached.

Analyses were performed using Stata 18 StataCorp, College Station, TX. Table 1 was made using the table 1 package in R, Version 2024.09.0 + 375. The regression models used to analyze the association between being satisfied with the check-up overall and the number of topics discussed were made in R. Predicted probabilities for being satisfied were calculated and visualized in R with the ggplot2 package.

**Table 1:** Descriptive characteristics of responders to survey about postpartum check-ups in Norway (2021–2022), by attendance

	Total	GP check-up	Midwife check-up	Hospital check-up	No check-up
	(*n* = 351)	(*n* = 211)	(*n* = 40)	(*n* = 3)	(*n* = 97)
	n (%)	n (%)	n (%)	n (%)	n (%)
**Age (years)**					
< 25	39 (11.1%)	16 (7.6%)	7 (17.5%)	0 (0%)	16 (16.5%)
25–29	140 (39.9%)	90 (42.7%)	16 (40.0%)	1 (33.3%)	33 (34.0%)
30–34	117 (33.3%)	72 (34.1%)	14 (35.0%)	0 (0%)	31 (32.0%)
≥ 35	55 (15.7%)	33 (15.6%)	3 (7.5%)	2 (66.7%)	17 (17.5%)
**Education**					
High school	117 (33.3%)	63 (29.9%)	14 (35.0%)	0 (0%)	40 (41.2%)
University 1–4 years	147 (41.9%)	85 (40.3%)	17 (42.5%)	2 (66.7%)	43 (44.3%)
University > 4 years	87 (24.8%)	63 (29.9%)	9 (22.5%)	1 (33.3%)	14 (14.4%)
**Parity**					
1	143 (40.7%)	87 (41.2%)	20 (50.0%)	0 (0%)	36 (37.1%)
2	142 (40.5%)	87 (41.2%)	15 (37.5%)	1 (33.3%)	39 (40.2%)
3	47 (13.4%)	29 (13.7%)	3 (7.5%)	0 (0%)	15 (15.5%)
4 or more	19 (5.4%)	8 (3.8%)	2 (5.0%)	2 (66.7%)	7 (7.2%)
**Self-rated health**					
Poor or not so good	29 (8.3%)	17 (8.1%)	2 (5.0%)	0 (0%)	10 (10.3%)
Good	108 (30.8%)	60 (28.4%)	11 (27.5%)	0 (0%)	37 (38.1%)
Very good	152 (43.3%)	98 (46.4%)	14 (35.0%)	3 (100%)	37 (38.1%)
Excellent	62 (17.7%)	36 (17.1%)	13 (32.5%)	0 (0%)	13 (13.4%)
**Physical chronic illness **^**a**^					
No	275 (78.3%)	169 (80.1%)	32 (80.0%)	2 (66.7%)	72 (74.2%)
Yes	76 (21.7%)	42 (19.9%)	8 (20.0%)	1 (33.3%)	25 (25.8%)
**Mental chronic illness **^**b**^					
No	315 (89.7%)	190 (90.0%)	36 (90.0%)	3 (100%)	86 (88.7%)
Yes	36 (10.3%)	21 (10.0%)	4 (10.0%)	0 (0%)	11 (11.3%)
**High-risk pregnancy **^**c**^					
No	283 (80.6%)	174 (82.5%)	33 (82.5%)	0 (0%)	76 (78.4%)
Yes	54 (15.4%)	29 (13.7%)	6 (15.0%)	3 (100%)	16 (16.5%)
Do not know	14 (4.0%)	8 (3.8%)	1 (2.5%)	0 (0%)	5 (5.2%)
**Labour complications **^**d**^					
No	226 (64.4%)	140 (66.4%)	24 (60.0%)	0 (0%)	62 (63.9%)
Yes	125 (35.6%)	71 (33.6%)	16 (40.0%)	3 (100%)	35 (36.1%)
**Mode of delivery**					
Vaginal	292 (83.2%)	180 (85.3%)	33 (82.5%)	2 (66.7%)	77 (79.4%)
C-section	59 (16.8%)	31 (14.7%)	7 (17.5%)	1 (33.3%)	20 (20.6%)
**Regular use of medication during pregnancy**					
No	259 (73.8%)	154 (73.0%)	32 (80.0%)	3 (100%)	70 (72.2%)
Yes	92 (26.2%)	57 (27.0%)	8 (20.0%)	0 (0%)	27 (27.8%)
**Municipality inhabitants**					
< 6000	57 (16.2%)	27 (12.8%)	8 (20.0%)	3 (100%)	19 (19.6%)
6000–20 000	142 (40.5%)	83 (39.3%)	16 (40.0%)	0 (0%)	43 (44.3%)
> 20 000	128 (36.5%)	87 (41.2%)	14 (35.0%)	0 (0%)	27 (27.8%)
Do not know	24 (6.8%)	14 (6.6%)	2 (5.0%)	0 (0%)	8 (8.2%)
**Know the check-up was free**					
No	111 (31.6%)	63 (29.9%)	9 (22.5%)	0 (0%)	39 (40.2%)
Yes	240 (68.4%)	148 (70.1%)	31 (77.5%)	3 (100%)	58 (59.8%)

*Abbreviation*: *GP* General Practitioner. C-section, cesarean section

^a^Physical chronic illness before pregnancy: High blood pressure, thyroid disease, diabetes, kidney disease, other

^b^Mental chronic illness before pregnancy: Depression, anxiety, post-traumatic stress disease, other

^c^Pregnancy defined as “high risk”

^d^Labour complications: Preeclampsia, major bleeding, cesarean section, baby in neonatal unit, preterm birth, other

## Results

### Characteristics of participants

Of 1,119 invited women, 351 (31.4%) responded. Characteristics of participants are demonstrated in Table 1. The largest age group was women 25 to 29 years, 41% were primiparous and 36% had labour complications. Regarding age, parity and education, the study participants seemed representative of women who gave birth in Trøndelag and Norway in 2021 (Supplementary Table 1s, Additional File 1). We note that the proportion of primipara, women under 25 years or living in smaller communities appeared to be higher among those who reported a check-up with a midwife compared to a GP.

More than half of the responders (52%) had received information about the postpartum check-up from a community midwife, 44% from the maternity ward, 20% from the GP, 18% from friends and 11% from social media. Other sources were indicated by 8%, while 5% had not received any information. Only 68% knew that the check-up was free of charge. Regarding the timing of the check-up, 282 (80.3%) reported that six weeks postpartum is an appropriate time, while 43 (12.3%) said it is too late, and 23 (6.6%) too early.

### Attendance

When asked if they had attended postpartum check-ups at the GP, 211 women responded confirmatively. Additionally, 40 women commented that they had a postpartum check-up with a midwife and three at a hospital, giving a total attendance of 254/351 (72%). The most common reasons for not attending were not having a regular GP (15 women), feeling it was unnecessary to attend (13 women), being unaware of the check-up (11 women) and not being satisfied with previous check-ups (10 women).

Total attendance was largely similar over demographic groups (Table 2), however, women with more than 4 years of higher education were more likely to attend (adjOR 2.9, CI 1.4–6.3) than those completing high school. Women with physical and mental chronic illness, high-risk pregnancies, regular use of medication during pregnancy, or complications during labour did not attend the check-up more frequently than healthy women with uncomplicated labour. Awareness that the check-up was free of charge increased the odds of attendance (adjOR 1.8, CI 1.1–2.9).

**Table 2:** Associations between attending a postpartum check-up and demographic and health predictors, among responders to survey about postpartum check-ups in Norway (2021- 2022)

Predictor	All respondents (*n* = 351)		All women with check-up (*n* = 254)	Odds ratio	95% CI	*p*-value
	n (%)		n (%)			
**Age (years)**						
< 25	39 (11.1%)		23 (9.1%)	0.51	(0.24—1.10)	0.08
25–29	140 (39.9%)		107 (42.1%)	1		
30–34	117 (33.3%)		86 (33.9%)	0.69	(0.38—1.24)	0.22
≥ 35	55 (15.7%)		38 (15.0%)	0.52	(0.25—1.09)	0.08
**Education**						
High school	117 (33.3%)		77 (30.3%)	1		
University 1–4 years	147 (41.9%)		104 (40.9%)	1.20	(0.69—2.06)	0.52
University > 4 years	87 (24.8%)		73 (28.7%)	2.90	(1.40—6.26)	< 0.01
**Parity**						
1	143 (40.7%)		107 (42.1%)	1		
2	142 (40.5%)		103 (40.6%)	0.78	(0.44—1.38)	0.40
3	47 (13.4%)		32 (12.6%)	0.62	(0.28—1.38)	0.24
4 or more	19 (5.4%)		12 (4.7%)	0.60	(0.21—1.86)	0.36
**Self-rated health**						
Excellent	62 (17.7%)		49 (19.3%)	1.10	(0.54—2.34)	0.80
Very good	152 (43.3%)		115 (45.3%)	1		
Good	108 (30.8%)		71 (28.0%)	0.66	(0.38—1.15)	0.14
Poor or not so good	29 (8.3%)		19 (7.5%)	0.67	(0.28—1.68)	0.38
**Physical chronic illness **^**a**^						
Yes	76 (21.7%)		51 (20.1%)	0.71	(0.40—1.26)	0.23
**Mental chronic illness **^**b**^						
Yes	36 (10.3%)		25 (9.8%)	0.87	(0.41—1.95)	0.72
**High-risk pregnancy **^**c**^						
Yes	54 (15.4%)		38 (15.0%)	0.94	(0.48—1.89)	0.86
**Labour complications **^**d**^						
Yes	125 (35.6%)		90 (35.4%)	1.07	(0.65—1.78)	0.79
**Mode of delivery**						
Vaginal	292 (83.2%)	215 (84.6%)		1		
C-section	59 (16.8%)	39 (15.4%)		0.78	(0.42—1.46)	0.43
**Regular use of medication during pregnancy**						
Yes	92 (26.2%)		65 (25.6%)	0.88	(0.51—1.52)	0.63
**Municipality inhabitants**						
> 20 000	128 (36.5%)		101 (39.8%)	1		
6000–20 000	142 (40.5%)		99 (39.0%)	0.64	(0.36—1.13)	0.13
< 6000	57 (16.2%)		38 (15.0%)	0.58	(0.29—1.19)	0.14
**Know that check-up is free**						
Yes	240 (68.4%)		182 (71.7%)	1.76	(1.06—2.92)	0.03
**Early contact with GP **^**e**^						
Yes	76 (21.7%)		57 (22.4%)	1.16	(0.65—2.14)	0.63

Each predictor was assessed in a separate logistic regression model, adjusted for age and education

*Abbreviation*: *CI* confidence interval, *GP* General Practitioner, *C-section* cesarean section

^a^Physical chronic illness before pregnancy: High blood pressure, thyroid disease, diabetes, kidney disease, other

^b^Mental chronic illness before pregnancy: Depression, anxiety, post-traumatic stress disease, other

^c^Pregnancy defined as “high risk”

^d^Labour complications: Preeclampsia, major bleeding, cesarean section, baby in neonatal unit, preterm birth, other

^e^Early contact with GP before the postpartum check-up

During the postpartum period, 76 women (22%) were in touch with their GP for other types of healthcare than a designated check-up. Physical consultations in the GP clinic were most frequent (*n* = 58), followed by phone calls (*n* = 15), written electronic consultations (*n* = 8), video consultations (*n* = 2) and home visit (*n* = 1). There was no significant difference in the likelihood of having a regular postpartum check-up between those with early contact with the GP and those without (adjOR 1.2, CI 0.7–2.1).

### Content of check-ups at the GP

**Fig. 1:**
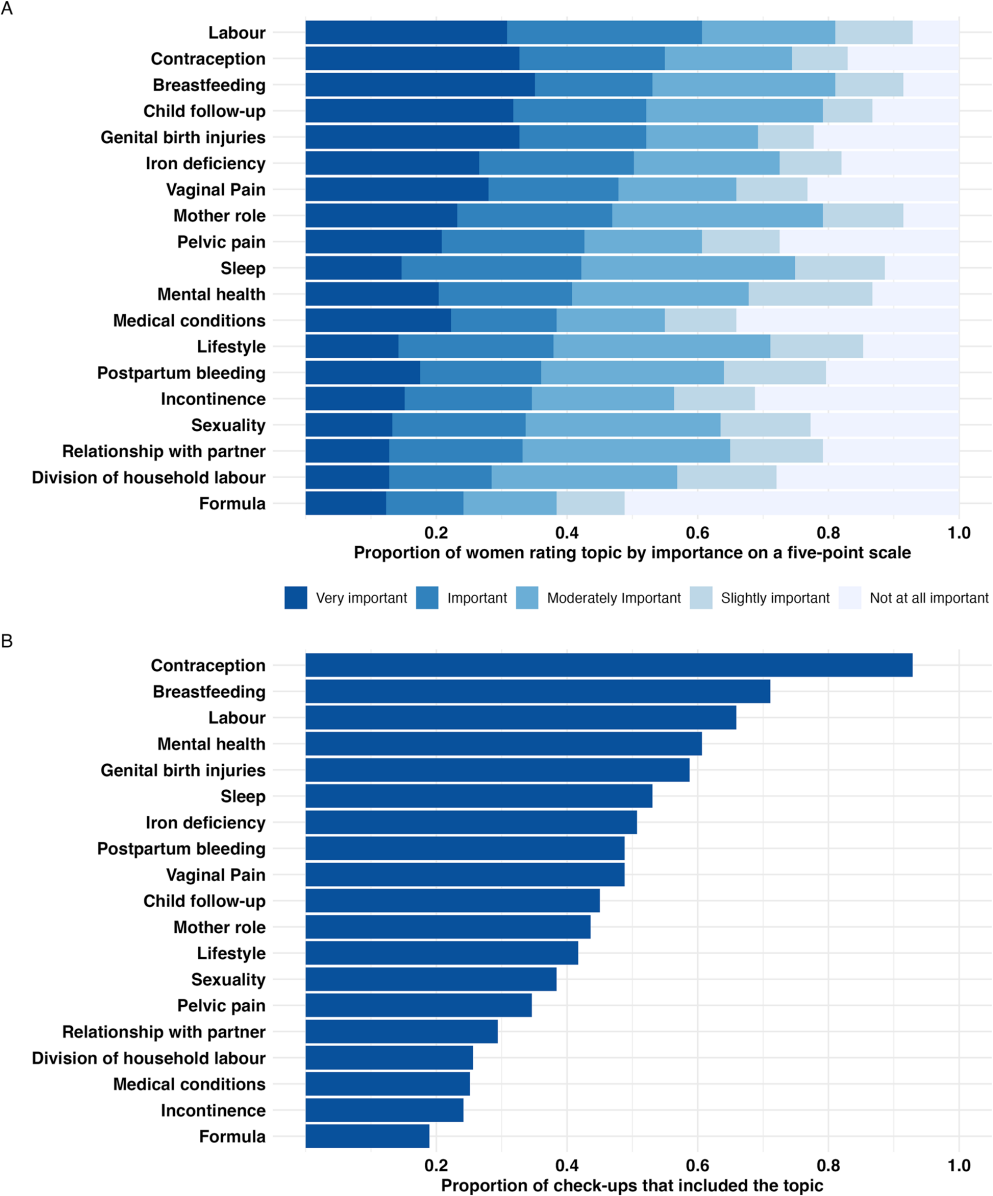
Importance and Coverage of Key Topics in Postpartum Check-ups with GPs (*N* = 211). **A** Proportion of women rating topic by importance**. (“**How important was this topic to you?”)**. B** Proportion of women who discussed topic during their postpartum check-up. (“ Did you discuss this topic?”)

Because this study focuses on care provided by GPs, only the 211 women who attended check-ups with GPs answered questions regarding importance of key topics, check-up content and satisfaction. The topics most frequently rated as “important” and “very important” were labour (61%), contraception (55%), breastfeeding (53%), genital birth injuries (52%) and follow-up of the child (52%) (Fig. 1A). Notably, all 19 topics were rated “important” or “very important” by more than 24.2% of the women. The topics most frequently discussed were contraception (93%), breastfeeding (71%), labour experiences (66%), mental health (61%), and genital birth injuries (59%) (Fig. 1B). The check-ups included a median of 8 topics (interquartile range (IQR) 5–12). Female GPs discussed a median of 9 (IQR 5–12) topics while male GPs discussed a median of 7 (IQR 5–11) topics (*p* = 0.07).

Clinical or laboratory examinations were performed in 80% of check-ups, including tests for anemia (51%), blood pressure (40%), gynecological examinations (34%), and abdominal examinations (28%). Women with female GPs had gynecological examinations more frequently than women with male GPs (42% vs. 23%, respectively).

### Patient satisfaction with GP check-ups

**Fig. 2:**
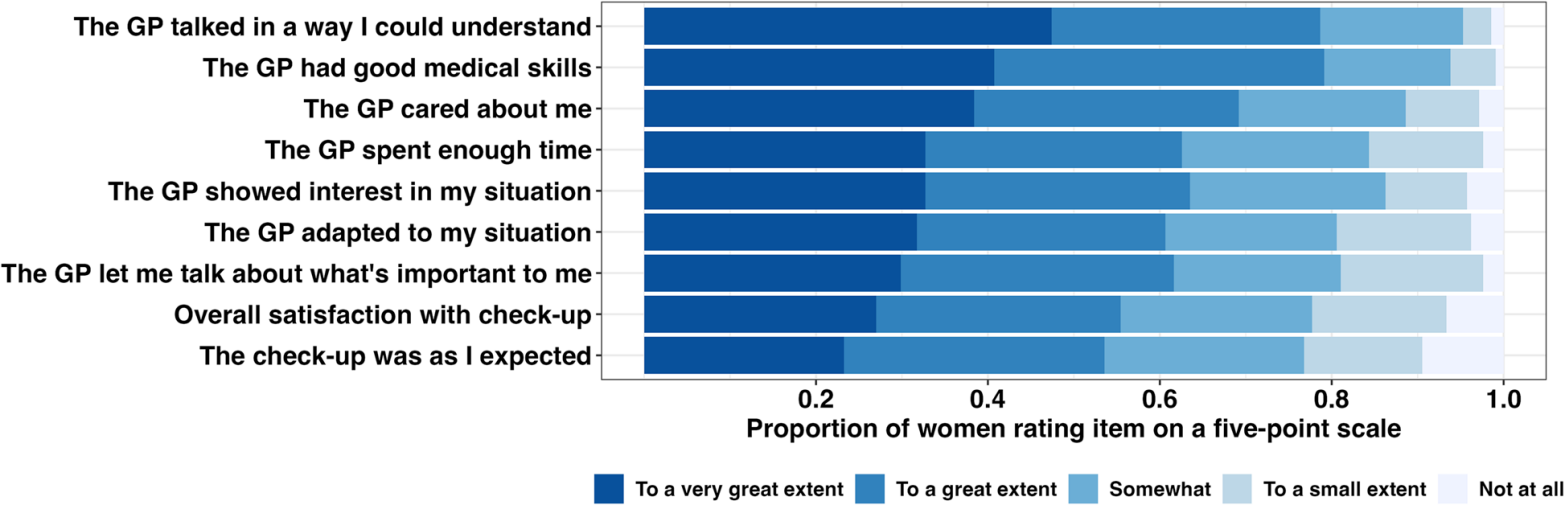
Patient reported experience items. Women who had a postpartum check-up with their GP rated their overall satisfaction and items regarding the GP on a five-point scale. *n* = 211

Women's rating of overall satisfaction and aspects regarding the GP are shown in Fig. 2. The highest ratings were given for the GP's ability to communicate clearly, demonstrate good medical skills, and show care for patients. For the dichotomous main outcome “Overall satisfaction with the check-up”, 117 women (55%) were satisfied. Additionally, 113 women (54%) indicated that the appointment met their expectations to a great or very great extent.

**Fig. 3:**
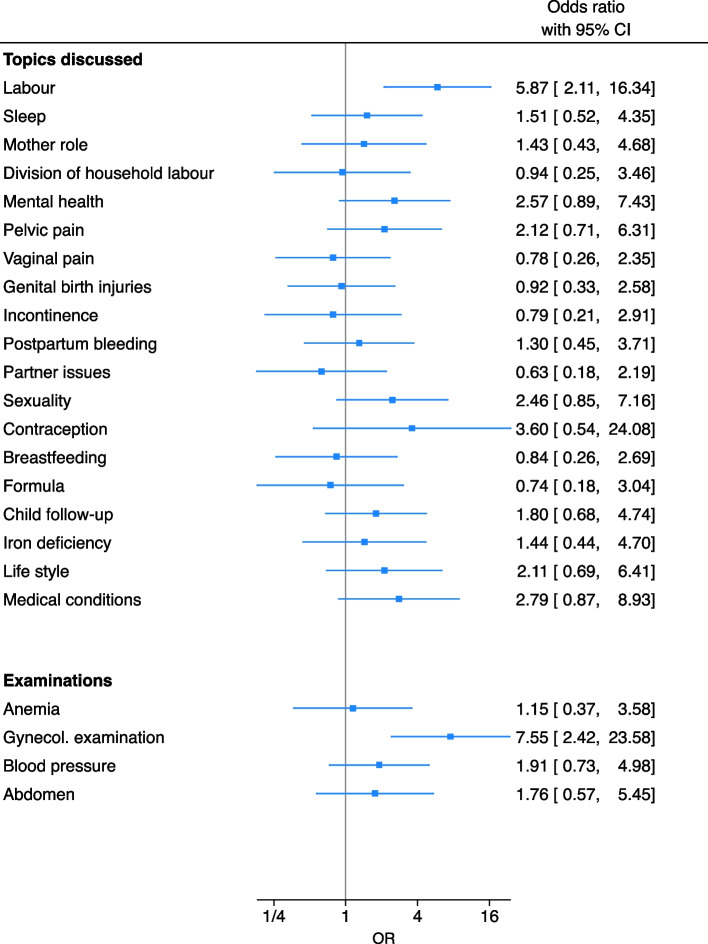
Association between being satisfied with the postpartum check-up and whether each key discussion topic and examination was covered. Logistic regression models adjusted for age, parity, physician gender and all other topics and examinations covered. Women who had postpartum check-ups with GPs. *n* = 211

The association between women being satisfied with the check-up overall (hereafter referred to as “being satisfied”), and whether each specific topic and examination was covered, is shown in Fig. 3. Discussing labour (adjOR 5.9, CI 2.1–16.3) and having a gynecological examination (adjOR 7.6, CI 2.4–23.6) were strongly associated with being satisfied.

Next, we examined the relationship between overall satisfaction and the number of topics discussed. The median number of topics discussed in the “satisfied” group was 9 (IQR 6–13), and in the “very satisfied” group 12 (IQR 10–16) (Supplementary Fig. 1s, Additional file 1). In the “not at all satisfied” group, the median number of topics covered was 4 (IQR 2–6).

We analyzed the relationship between being satisfied and the number of topics discussed, adjusting for gynecological examination in logistic regression (model 2, supplementary Table 2s, Additional File 1). For each additional topic discussed, the odds of satisfaction increased by 38% (adjOR 1.38, CI 1.3—1.5), holding gynecological examination constant. There was an almost fourfold increase in the odds of satisfaction (adjOR 3.96, CI 1.9—8.6) when a gynecological examination was performed, holding the number of topics constant.

Women with three or four births reported higher overall satisfaction than women with one or two births. Due to potential selection bias among multipara, a sensitivity analysis restricted to women with one or two births was conducted, giving even higher odds ratios for the number of topics and for the gynecological examination (Table 2s, model 6, Additional file 1). Abdominal examinations and blood pressure measurements were not significantly associated with being satisfied (Table 2s, model 7 and 8, Additional file 1).

**Fig. 4:**
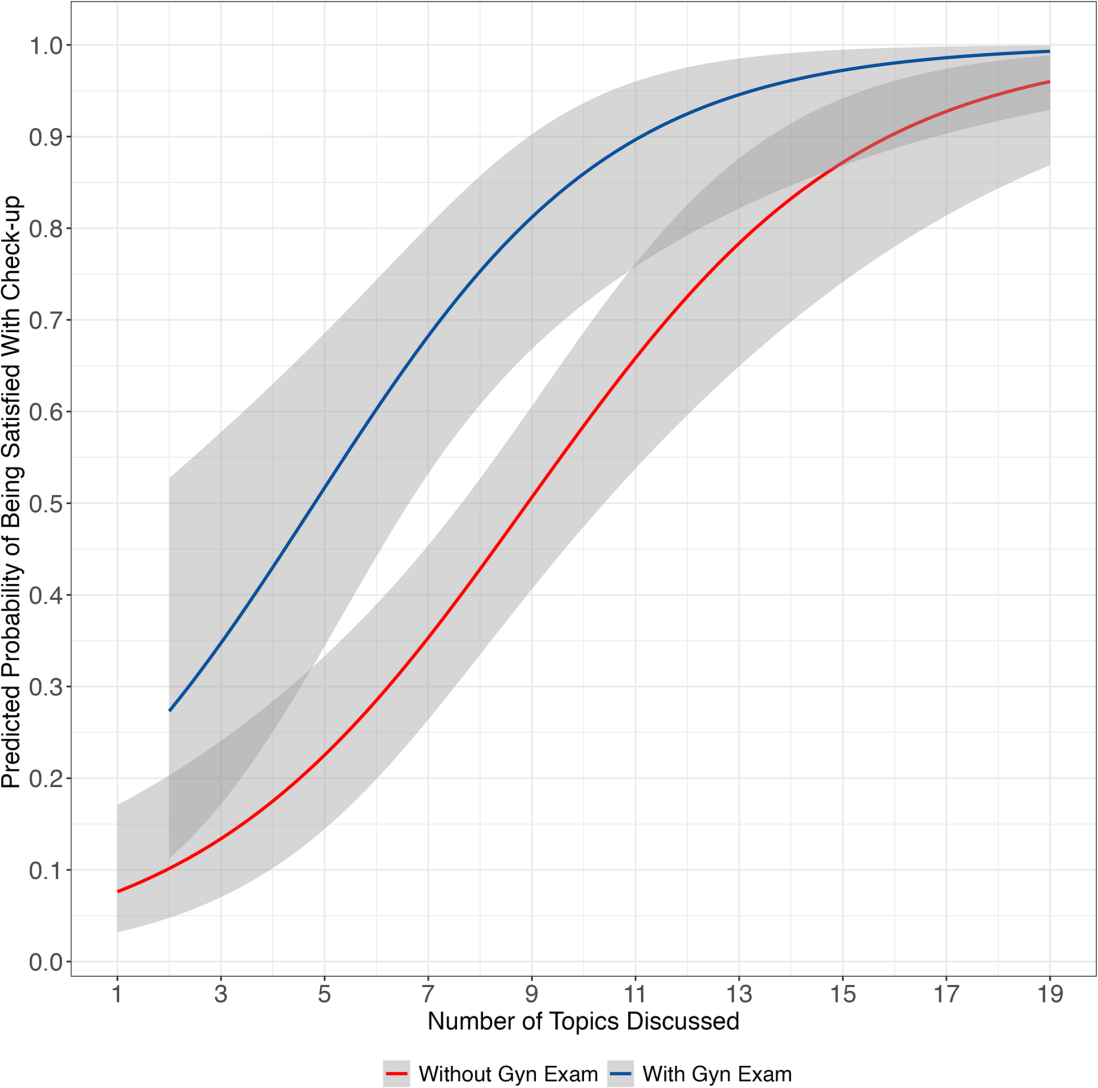
Predicted probability of being satisfied with the postpartum check-up with the GP by number of topics discussed, with and without a gynecological examination. GP = general practitioner. Gyn exam = gynecological examination. *n* = 211

Next, we predicted the probabilities of being satisfied by the number of topics discussed, with and without a gynecological examination, plotted in Fig. 4. When discussing five topics, the predicted probability of being satisfied is 22% without a gynecological examination. However, if the gynecological examination is performed the probability of being satisfied is 53%. When discussing 10 topics, the predicted probability of being satisfied is 59% without a gynecological examination and 85% with it.

We examined the association between physician gender and overall satisfaction. Using univariate logistic regression, women who had female GPs were more satisfied with the check-up than those who had male GPs (OR 2.2, CI 1.2–3.8). However, the association was no longer significant when adjusting for the number of topics and examinations (adjOR 1.9, CI 0.9–3.7).

A total of 95 women provided free-text suggestions for improvement. Among these, 23 recommended that all women should be offered a gynecological examination, 23 suggested allowing more time, while 20 proposed a more structured conversation covering important and potentially embarrassing topics. Additionally, two women suggested implementing an automatic summon, and two others recommended having more than one check-up.

## Discussion

Of the women in the study, 72% attended a postpartum check-up with either a GP, community midwife or in the hospital. Attendance did not differ by parity, chronic disease or adverse pregnancy outcomes. The most frequent reasons for not attending were lack of a regular GP, feeling the check-up was unnecessary, low satisfaction with previous check-ups and lack of knowledge about check-ups. The most important discussion topics identified by women were labour experiences, contraception, breastfeeding and genital birth injuries. These topics, along with mental health were also the most frequently covered. Consultations covered a median of eight discussion topics. Clinical or laboratory examinations were performed for 80% of the women, but only 34% had a gynecological examination. Most women who saw a GP were satisfied with the care received. Satisfaction was strongly related to the number of topics discussed, conversations about labor experiences, and gynecological examinations. Women suggested GPs should structure comprehensive conversations covering many topics, allocate more time, and offer gynecological examinations.

### Attendance

The attendance rate in our study (72%) is comparable to the mean rate across 88 U.S. studies [16] but lower than the 77–90% rates in UK and Swedish studies [15, 17, 18]. According to women, attendance could increase if they have regular GPs, as they prefer care from GPs who know them. Only 44% of the respondents reported receiving information about the check-up from the maternity ward, although all mothers should be informed before discharge [10]. Similarly, a U.S. study found most women could not recall a postpartum primary care recommendation from their pregnancy care team [34], and others received inconsistent information about the timing and purpose of check-ups [10]. Attendance could be improved by structured discharge planning, written information about the timing, purpose and free availability of check-ups, digital invitations and reminders from GPs, and improved quality of check-ups.

Women with preexisting conditions and adverse pregnancy outcomes did not attend the postpartum check-up more frequently than those in good health, which is concerning given their increased risk of morbidity and mortality [35–37]. This aligns with previous findings of low attendance among women with depression [38] and those with adverse pregnancy outcomes [39]. Conversely, women with preexisting chronic conditions were more likely to have postpartum health care visits in some studies [39, 40].

In our study, 32% of the responders were unaware that the check-up is free of charge. Since knowing the check-up is free was associated with higher attendance, increasing awareness about full coverage could potentially improve attendance.

### Check-up content and satisfaction with check-ups with GPs

The topics most important to women were frequently addressed during check-ups, indicating that GPs either share similar perceptions of important topics or effectively adapt to women’s concerns and priorities. Labour was the most important topic according to women and was discussed in 66%. Overall satisfaction was strongly associated with discussing labour, aligning with other studies that highlight the importance of processing childbirth [8, 41, 42]. Contraception was discussed in approximately 90% of consultations, compared to 57–88% of check-ups in the U.S. [25, 43]. Breastfeeding was frequently addressed, which is crucial given its high importance and well-documented benefits for mothers, children and public health [44]. Support increases the likelihood of mothers meeting their breastfeeding goals [45]. Mental health was discussed in over 60% of check-ups, while 41% of women identified it as important/very important. This may indicate that physicians actively raise the topic recognizing its significance, even though formal screening is not recommended in Norway [23]. This finding is particularly relevant given that 32% mothers had high scores for depressive symptoms in a 2021 Norwegian study [46]. Mental health was addressed with similar frequency as U.S. Family Physicians [25], whereas 93% of GPs in an English study reported that they always or very often discuss mental health [12].

Pain [15], genital injuries [47], incontinence [48] and sexual problems [49] are common after childbirth. In our study, genital birth injuries and pain were among the most important and frequently discussed topics. Despite this, only 34% of women in our study had a gynecological examination. Comparably, only 18% of English GPs always or very often examined women with vaginal problems [12]. In contrast, 75% of women in a Swedish study had vaginal examinations at postpartum check-ups [15]. An American study found that providers conducted gynecological examinations more frequently than they deemed necessary [19], suggesting a discrepancy in perceived importance between physicians and women. NICE states that perineal pain is often falsely seen as part of normal postpartum healing, and should be assessed and managed early, recommending examinations if there are concerns about healing or if the woman seeks reassurance [2]. This recommendation seems well aligned with women’s opinions: The odds of being satisfied with the check-up were substantially higher when a gynecological examination was conducted. Several women suggested that GPs should offer the gynecological examination proactively, as many felt uncomfortable requesting it. Given these findings, it is likely that gynecological examinations were warranted in more than 34% of the check-ups and should be offered more often.

The lowest ratings were related to the time GPs spent during consultations and their ability to adapt to individual needs. American and Australian GPs reported spending 25–30 min per postpartum visit [10, 19] and English GPs consider the allocated consultation time insufficient [12]. The average duration of all GP consultations in Norway is 18 min [50]. Strengthened incentives for GPs to provide comprehensive care, for example higher reimbursement for longer consultations or for postpartum check-ups, could increase quality and satisfaction. There is an ongoing shift in the U.S. from a single check-up to a series of postpartum care visits [3]. An increase from one to two check-ups has been suggested in Norway [51]. This could facilitate coverage of more concerns and health promotion.

Since women’s health and priorities vary, tailoring the check-up to each woman’s health needs while actively bringing up important, possibly sensitive topics, could increase quality and satisfaction. The American College of Obstetricians and Gynecologists endorses the use of a structured checklist [52]. GPs in England who used a template for support more frequently delivered clinical activities recommended by NICE, and had longer consultations [12]. In the Norwegian context, the use and impact of support tools such as patient preparation forms or GP checklists warrant further exploration, particularly regarding how they influence the balance between patient-centered care and check-list driven consultations.

Women in our study were generally satisfied with the communication, care and medical skills of the GPs, although even higher levels of satisfaction have been reported from Norwegian obstetric and somatic wards [21, 31]. Higher overall satisfaction among multiparous women could be due to selection bias, as women who were more satisfied with their previous check-ups might be more likely to attend again. Still, this potential bias did not reduce the importance of the number of topics or gynecological examinations, shown by the sensitivity analysis restricted to women with one and two children. Higher satisfaction with female GPs seemed to be due to gender differences in check-up content, also found in England, where female GPs more frequently discussed birth experience, pelvic, bladder and breast problems, wound healing, mental well-being and examined vaginal problems and perineal wounds [12]. These gender differences may stem from GPs’ personal experiences as mothers, which can increase their understanding of mothers’ postpartum needs. GPs who were parents also delivered more clinical items from the NICE guidelines compared to GPs without children [12], suggesting a correlation with personal experience. Enhanced training and support tools like clear guidelines could improve the quality of check-ups, regardless of GPs’ gender and personal experiences.

### Strengths and limitations

Since the survey was designed to address postpartum check-ups performed by GPs, we might have underestimated the number of check-ups performed by midwives and thereby the total attendance. The participation rate of 31.4% is a potential limitation. Still, as the distribution of age, parity, and education among the respondents resembles that of Norwegian mothers, the results should be relevant for women and physicians in Norway and similar health care systems. Data were self-reported and collected weeks after the check-up, which might introduce recall bias. However, maternal reporting of birth and pregnancy information generally has high validity [53]. In contrast to gynecological examinations, other examinations were not associated with higher satisfaction; however, the estimates were imprecise. While the correlation between gynecological examinations and satisfaction was robust, causation cannot be established with this study design. Unmeasured factors, such as the duration of the check-up, could also contribute to the observed satisfaction levels. A strength of the study is its comprehensive mapping of the check-up content, rather than focusing on one or a few components. Another strength is that almost all women who gave birth to living children in the region during a whole year were invited. As Norway is a high-income country with a well-developed health care system, generally good public health and low maternal mortality, the results may be less generalizable to countries with different settings and disease burdens.

## Conclusion

Fewer than three of four women attended the postpartum check-up. It is particularly concerning that women with pre-existing conditions and adverse pregnancy outcomes did not attend more frequently than those in good health. Access to a regular GP, improved quality of check-ups, and informing all mothers about check-ups could increase attendance.

The majority of women were satisfied with the postpartum check-up with the GP. Discussing a broad range of topics, including the labour experience and offering gynecological examinations could improve patient satisfaction further. The broad scope suggests that sufficient time should be allocated. The postpartum check-up should be tailored to each woman’s needs, aiming to enhance health and well-being, not only during the postpartum period but also from a lifelong perspective.

## Supplementary Information

Supplementary Material 1

## Data Availability

The dataset is available from the corresponding author on reasonable request.

## Abbreviations

adjOR: Adjusted odds ratio
AIC: Akaike Information Criterion
CI: Confidence interval
GP: General practitioner
HNT: Nord-Trøndelag Health Trust
IQR: Interquartile range
NICE: National Institute for Health and Care Excellence
OR: Odds ratio
UK: United Kingdom
U.S.: United States
WHO: World Health Organization
