# Supplementary material for: Postpartum check-ups with general practitioners in Norway: a cross-sectional survey of attendance, content and patient satisfaction
Source: BMC Prim Care. 2025 Oct 7;26:306. doi: 10.1186/s12875-025-02992-x (PMC12506350; doi:10.1186/s12875-025-02992-x)
Supplement: Supplementary file 1 — Supplementary Material 1 [file 12875_2025_2992_MOESM1_ESM.pdf]

Supplementary tables, figures and questionnaire:

***Table 1s: Age, parity and education in study respondents and women who gave birth in Trondelag county and Norway in 2021.***

| | Study | Trondelag county [a] | Norway [a,b] |
|---|---|---|---|
| **Age** | | | |
| < 25 | 11.1% | 9% | 7.6% |
| 25-29 | 39.9% | 34% | 30% |
| 30-34 | 33.3% | 37.6% | 40.1% |
| 35 or over | 15.7% | 19.4% | 22.3% |
| **Parity** | | | |
| 1 | 40.7% | 42.3% | 42.5% |
| 2 | 40.5% | 37.4% | 37.5% |
| 3 | 13.4% | 14.8% | 14.4% |
| 4 or more | 5.4% | 5.5% | 5.6% |
| **Education** | | | |
| High school | 33.3% | | 41.3% |
| University 1-4 years | 41.9% | | 39.4% |
| University > 4 years | 24.8% | | 18.7% |

[a] Age and parity of women giving birth in 2021, data from Medical Birth Registry of Norway.
[b] Education level, weighted to the age distribution of women who gave birth in Norway in 2021, data from Statistics Norway.

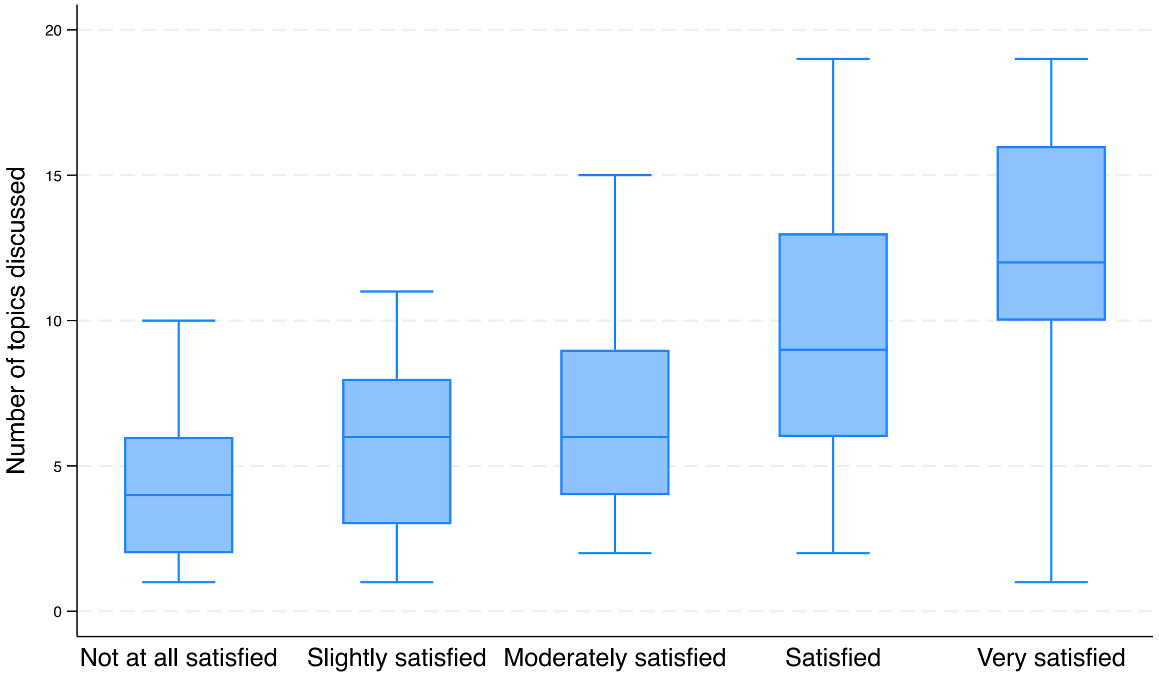


**Figure 1s: Overall satisfaction with the postpartum check-up with the GP by number of topics discussed.**
Median number of topics discussed in each satisfaction level, with interquartile range and ranges for top and bottom 25% of the data values. n=211. Norway, 2021-2022

*Table 2s: Association between being satisfied with the check-up and the total number of topics discussed, adjusted for different examinations.*

| Model and covariates | OR # of topics | 95% CI | p-value topics | OR gyn ex | 95% CI | p-value gyn ex | AIC |
|---|---|---|---|---|---|---|---|
| M1: Number of topics | 1.37 | (1.26 - 1.52) | < 0.001 | | | | 224.2 |
| **M2: Number of topics + gyn ex** | **1.38** | **(1.26 - 1.54)** | **< 0.001** | **3.96** | **(1.9 - 8.63)** | **< 0.001** | **212.2** |
| M3: M2 + BP + abdominal ex | 1.36 | (1.24 - 1.52) | < 0.001 | 3.75 | (1.77 - 8.32) | < 0.001 | 214.9 |
| M4: M3 + parity + age | 1.44 | (1.29 - 1.63) | < 0.001 | 5.41 | (2.33 - 13.46) | < 0.001 | 194.8 |
| M5: M4 + GP duration + GP gender | 1.43 | (1.28 - 1.61) | < 0.001 | 4.73 | (2 - 11.91) | < 0.001 | 195.8 |
| M6: M2 (only para 1 and 2) | 1.44 | (1.3 - 1.63) | < 0.001 | 4.42 | (1.95 - 10.58) | < 0.001 | |

| Model | OR # of topics | 95% CI | p-value topics | OR ex | 95% CI | p-value ex | AIC |
|---|---|---|---|---|---|---|---|
| M7: M1 + abdomen | 1.35 | (1.21 - 1.40) | < 0.001 | 1.89 | (-0.13 – 1.43) | 0.11 | 223.6 |
| M8: M1 + BP | 1.36 | (1.22 - 1.41) | < 0.001 | 1.40 | (-0.35 – 1.02) | 0.34 | 225.3 |

Logistic regression models. Outcome: Overall satisfaction with check-up.
OR; odds ratio. #; number. CI; confidence interval. Gyn; gynecological. Ex; examination. BP; blood pressure. GP; general practitioner. AIC; Akaike Information Criterion.

# English translation of Questionnaire:

Experiences with pregnancy and postpartum check-ups at the general practitioner (GP)

**Background Questions**
**1. Age:** What is your age? (<25, 25-29, 30-34, 35-39, >40)
**2. What is your highest completed education?** (Elementary school, high school, university/college 1-4 years, university/college over 4 years)
**3. Were you born in Norway?** Yes/No
**4. How many inhabitants are there in the municipality you live in?**
(<2000, 2000-6000, 6000-20,000, 20,000-50,000, over 50,000)
**5. How long have you had your current GP?**
Number of years, Locum/Substitute, No GP
**6. What is the gender of your GP?** Female, Male
**7. Overall, would you say your health is...**
Excellent - Very good - Good - Fairly good - Poor
**8. How many times have you given birth?** One – Two – Three – Four or more

If you have given birth multiple times, please answer the following questions based on your experiences with your most recent pregnancy, birth, and postpartum period.

**9. Was the pregnancy classified as a high-risk pregnancy?** Yes, No, Don’t know

**10. Did you have any chronic illnesses before you became pregnant?** Checkboxes:
- High blood pressure
- Thyroid disease
- Diabetes
- Kidney disease
- Depression
- Anxiety
- Post-traumatic stress disorder
- Arthritis
- Other

**11. Did you experience any of the following during childbirth?** Checkboxes:
- Preeclampsia
- Major bleeding requiring surgery or blood transfusion
- Planned cesarean section
- Emergency cesarean section
- Baby needed monitoring/treatment in the neonatal unit
- Birth before 37 weeks of pregnancy
- Other

**12. Did you take regular medication <u>before</u> pregnancy?** Yes/No
**13. Did you take regular medication <u>during</u> pregnancy?** Yes/No

**14. How many prenatal check-ups did you have with your GP?**
None, 1-4, 5-9, 10-16, 17 or more
**15. How many prenatal check-ups did you have with a midwife?**
None, 1-4, 5-9, 10-16, 17 or more
**16. Was your first prenatal check-up with a GP or a midwife?**
**17. Did you have prenatal check-ups at the hospital or with a gynecologist outside of routine ultrasound?** Yes/No

**18. How long was your postpartum hospital stay after childbirth?**
Less than 24 hours – 1-2 days – 3-4 days – 5-6 days - 7 days or more
**19. Do you think the length of your postpartum hospital stay was appropriate?**
Yes – No, too short – No, too long If no, why? Open text box

## Experiences with <u>Prenatal</u> Care

If you had prenatal check-ups with your GP:

**20. Did you get to talk to your GP about what you were concerned about?**
Not at all - To a small extent - To some extent - To a large extent - To a very large extent

If the answer to the previous question is: not at all, to a small extent, or to some extent:
**21. What would you have liked to discuss?** Open text box.

Questions 22-29 have the following response options:
Not at all - To a small extent - To some extent - To a large extent - To a very large extent

**22. Did the doctor talk to you in a way that you understood?**
**23. Did you feel that the doctor cared about you?**
**24. Do you trust the doctor's professional competence?**
**25. Did the doctor have time for you when you needed it?**
**26. Did you get to tell the doctor everything you thought was important about your condition?**
**27. Did you feel that the doctor was interested in your description of your situation?**
**28. Did you feel that the follow-up and treatment were tailored to your situation?**
**29. How was your overall satisfaction with the prenatal check-up with your GP?**

**30. What would have made you more satisfied with the prenatal check-ups with your GP?** Open text box

**31. Did you and your doctor discuss postpartum depression before childbirth?** Yes/No
**32. Did you and your doctor discuss physical activity and diet during the prenatal check-up?** Yes/No

If you had prenatal check-ups with a midwife:
**33. How satisfied are you overall with the prenatal check-ups with the midwife?**
Not at all - To a small extent - To some extent - To a large extent - To a very large extent

## Experiences with Postpartum Care

After childbirth, it is recommended to have a check-up for the mother 4-6 weeks after birth. This check-up is often called the 6-week check-up, postpartum check-up, control or visit. In this survey, we call it the "postpartum check-up".

The consultation should focus on the woman's physical and mental health and include information about contraception. The purpose is both to identify issues that need follow-up and to support the woman.

**34. How did you learn about the postpartum check-up for the mother?**
From GP, community midwife, maternity ward, friend, other (free text field), did not know. (Multiple choices allowed)
**35. Did you know that a postpartum check-up is free?** Yes, No
**36. Were you in contact with your GP before the postpartum check-up?**
No, Yes in the 1st week, Yes in the 2nd week, Yes in the 3rd-4th week, Yes in the 4th-6th week

**37. If yes, what kind of contact?**

- Phone
- Video consultation
- E-consultation
- Consultation at the doctor's office
- Home visit from the doctor

(Multiple choices allowed)

**38. If yes to question 36 – what was the reason for contacting your GP**? Open text box

**39. Did you have to return to the maternity ward at the hospital after discharge?**
Yes/No. If yes, why?

**40. Did you feel that the transition between the hospital and follow-up in the community (GP, midwife, health station) was safe?**
Not at all, To a small extent, To some extent, To a large extent, To a very large extent

**41. Have you had a postpartum check-up with your GP?**
Yes/No.
**If no, why not?** Open text box

## Experiences and Content of Postpartum Check-up with the GP:

**If you had a postpartum check-up with your GP** (yes in question 41)**:**

**42. Did you get to talk to your GP about what you were concerned about?**
Not at all - To a small extent - To some extent - To a large extent - To a very large extent

**The conversation with the doctor**
**43. Was this topic discussed?** No Yes
**44. How important was this topic to you?**
0 not at all important – 1 – 2 – 3 – 4 very important

- Course of childbirth/labour
- Sleep and rest
- Motherhood
- Division of household work with partner
- Low mood
- Worry and rumination
- Postpartum depression
- Pelvic pain
- Genital pain
- Wounds/lacerations in the perineum and healing
- Urinary incontinence
- Lochia/bleeding
- Relationship with partner
- Sexuality
- Contraception
- Breastfeeding
- Infant formula
- Follow-up of the child
- Blood loss, iron deficiency
- Diet
- Physical activity/exercise

- Follow-up of illnesses in you

**45. If other topics were raised by the doctor – which ones?** Open text box
**46. Did you bring up other topics yourself?** Yes/No.
If yes – which ones? Open text box

**47. Are there other topics you think should be discussed at the 6-week check-up?**
Open text box

**48. What examinations were done?**

- Check of hemoglobin/iron stores
- Gynecological examination
- Blood pressure
- Abdominal examination
- Other?

**49. Were there other examinations you wished you had?** Open text box

**Satisfaction with Postpartum Check-up**
Response options for questions 50-56 are:
Not at all, To a small extent, To some extent, To a large extent, To a very large extent

**50. Did the doctor talk to you in a way that you understood?**
**51. Did you feel that the doctor cared about you?**
**52. Do you trust the doctor's professional competence?**
**53. Did the doctor have time for you when you needed it?**
**54. Did you get to tell the doctor everything you thought was important about your condition?**
**55. Did you feel that the doctor was interested in your description of your situation?**
**56. Did you feel that the treatment was tailored to your situation?**
**57. How satisfied are you overall with the postpartum check-up with your GP?**
**58. Overall, was the service you received at the postpartum check-up as expected?**

**59. What would make you more satisfied with the postpartum check-up with your GP?** Open text box

**60. The timing of the postpartum check-up (6 weeks after birth) - is it**
Too late – Just right – Too early

**61. Looking back at the prenatal check-ups – is there anything you now wish you had more information about during the prenatal check-up with your GP?**
Open text box
